# Immunosuppression Has Long-Lasting Effects on Circulating Follicular Regulatory T Cells in Kidney Transplant Recipients

**DOI:** 10.3389/fimmu.2020.01972

**Published:** 2020-08-28

**Authors:** Qian Niu, Aleixandra Mendoza Rojas, Marjolein Dieterich, Dave L. Roelen, Marian C. Clahsen-van Groningen, Lanlan Wang, Teun van Gelder, Dennis A. Hesselink, Nicole M. van Besouw, Carla C. Baan

**Affiliations:** ^1^Department of Laboratory Medicine, West China Hospital, Sichuan University, Chengdu, China; ^2^The Rotterdam Transplant Group, Department of Internal Medicine–Nephrology & Transplantation, Erasmus MC, University Medical Center Rotterdam, Rotterdam, Netherlands; ^3^Rotterdam Transplant Group, Erasmus MC, University Medical Center Rotterdam, Rotterdam, Netherlands; ^4^The Rotterdam Transplant Group, Department of Clinical Pharmacology, University Medical Center Rotterdam, Rotterdam, Netherlands; ^5^Department of Immunohematology and Blood Transfusion, Leiden University Medical Center, Leiden, Netherlands; ^6^The Rotterdam Transplant Group, Department of Pathology, Erasmus MC, University Medical Center Rotterdam, Rotterdam, Netherlands

**Keywords:** kidney transplantation, antibody mediated rejection, anti-rejection therapy, donor specific antibodies, flow cytometry, circulating Tfr, circulating Tfh, transplantation immunology

## Abstract

**Background:** FoxP3^+^ follicular regulatory T cells (Tfr) have been identified as the cell population controlling T follicular helper (Tfh) cells and B cells which, are both involved in effector immune responses against transplanted tissue.

**Methods:** To understand the biology of Tfr cells in kidney transplant patients treated with tacrolimus and mycophenolate mofetil (MMF) combination immunosuppression, we measured circulating (c)Tfh and cTfr cells in peripheral blood by flow cytometry in *n* = 211 kidney transplant recipients. At the time of measurement patients were 5–7 years after transplantation. Of this cohort of patients, 23.2% (49/211) had been previously treated for rejection. Median time after anti-rejection therapy was 4.9 years (range 0.4–7 years). Age and gender matched healthy individuals served as controls.

**Results:** While the absolute numbers of cTfh cells were comparable between kidney transplant recipients and healthy controls, the numbers of cTfr cells were 46% lower in immunosuppressed recipients (*p* < 0.001). More importantly, in transplanted patients, the ratio of cTfr to cTfh was decreased (median; 0.10 vs. 0.06), indicating a disruption of the balance between cTfr and cTfh cells. This shifted balance was observed for both non-rejectors and rejectors. Previous pulse methylprednisolone or combined pulse methylprednisolone + intravenous immunoglobulin anti-rejection therapy led to a non-significant 30.6% (median) and 51.2% (median) drop in cTfr cells, respectively when compared to cTfr cell numbers in transplant patients who did not receive anti-rejection therapy. A history of alemtuzumab therapy did lead to a significant decrease in cTfr cells of 85.8% (median) compared with patients not treated with anti-rejection therapy (*p* < 0.0001). No association with tacrolimus or MMF pre-dose concentrations was found.

**Conclusion:** This cross-sectional study reveals that anti-rejection therapy with alemtuzumab significantly lowers the number of cTfr cells in kidney transplant recipients. The observed profound effects by these agents might dysregulate cTfr functions.

## Introduction

Improvement of long-term outcomes after kidney transplantation remains a challenge (1–5). The most recent findings based on the United States registry data and Collaborative Transplant Study across 21 European countries, report only a slight improvement in renal allograft survival since the early 2000's (6–8). Antibody-mediated immune responses are recognized as an important factor in late kidney allograft failure (9–11). This immune response is refractory to treatment with conventional immunosuppression (12–14).

Follicular T helper (Tfh) cells play a critical role in B cell-dependent antibody generation (15–17). These Tfh cells co-localize with B cells in germinal centers within secondary lymphoid organs (SLOs) and are specialized in assisting antigen activated B cells to differentiate into antibody-producing plasma cells (18, 19). This immune response is controlled by follicular regulatory T (Tfr) cells, a unique subset of regulatory T (Treg) cells, that inhibits Tfh and B cell responses (20, 21). Tfr cells exert immune inhibitory functions through down-regulating the co-stimulatory molecule CD86 on B cells (22), producing inhibitory cytokines e.g., interleukin (IL)-10 and mediating cytolysis (23). Both Tfr and Tfh cells express high levels of CXC chemokine receptor 5 (CXCR5) and programmed death 1 (PD-1) (24), as well as the transcriptional factor B cell lymphoma (Bcl)-6 (25, 26). Tfr cells can also express Helios a transcription factor expressed by natural Tregs, and a marker reflecting enhanced immunosuppressive functions (27). From studies in patients suffering from auto-immune disease we know that the dynamic balance between Tfr and Tfh cells is important for immune homeostasis and tolerance. An aberrant Tfr/Tfh ratio has been linked to the development of autoantibody immunity-mediated diseases including systemic lupus erythematosus (28), rheumatoid arthritis (29), and myasthenia gravis (30). Also in kidney transplant recipients it was shown that antibody mediated rejection (ABMR) and chronic allograft dysfunction are associated with a disturbed balance of circulating (c)Tfh and cTfr cells (31, 32).

The role of Tfh cells in alloreactivity was shown by de Graav et al. (33) and de Leur et al. (34) who found that Tfh cells are present in biopsies diagnosed as T cell-mediated rejection (TCMR) and ABMR and that these Tfh cells provide help to alloantigen-activated B cells (35). From experimental transplant models we know that Tfh cells are capable of stimulating alloantibody production which in turn mediates the humoral response against the allograft (36, 37). These findings highlight the importance of Tfh cells in the process of transplant rejection while the first data about Tfr cells point to their impaired function in controlling the actions of Tfh cells in the allogeneic responses after organ transplantation (32).

Immunosuppressive drugs, including the calcineurin inhibitor (CNI) tacrolimus, may decrease the number and impair the function of Tfh and Tfr cells (38). However, to what extent immunosuppressive therapies affect the biology of Tfr and Tfh cells after kidney transplantation is unknown.

In the present observational study, the absolute numbers of Tfr cells and their relation to Tfh cells in immunosuppressed renal transplant recipients and healthy controls was investigated. In addition, the impact of anti-rejection therapy on these cell populations was studied.

## Materials and Methods

### Study Population

A cohort of 211 renal transplant recipients transplanted between 2012 and 2014 was sampled cross sectionally at the timepoint of 5.0–7.7 years (median 5.3) post-transplant. All patients had a functional graft at the time of blood sampling, kidney function was assessed by estimated glomerular filtration rate (eGFR, mL/min per 1.73 m^2^) as calculated by the CKD-EPI equation. Transplant recipients (*n* = 202) received induction therapy with basiliximab [Simulect® Novartis, Basel, Switzerland; 20 mg intravenously on days 0 and 4], rabbit anti-thymocyte globulin [Thymoglobulin®, Sanofi Genzyme, United States (*n* = 2) or rituximab [MabThera® Roche, Basel, Switzerland (*n* = 7) for blood group ABO incompatible kidney transplantation [rituximab 375 mg/m^2^ 4 weeks before transplantation; tacrolimus 0.1 mg/kg *b.i.d*, mycophenolate mofetil (MMF) 1,000 mg *b.i.d*; prednisone 20 mg once daily starting 2 weeks before transplantation]. The post-operative immunosuppressive regimen after transplantation consisted of tacrolimus (Prograf® Astellas Pharma, Tokyo, Japan; aiming for pre-dose concentrations of 10–15 ng/mL in weeks 1–2, 8–12 ng/mL in weeks 3–4 and 5–10 ng/mL thereafter), MMF (Cellcept® Roche, Basel, Switzerland; starting dose of 1 g twice a day, aiming for pre-dose concentrations of 1.5–3.0 mg/L) and prednisolone. Prednisolone was tapered to 5 mg at month 3 and withdrawn at months 4–5. In patients who experienced a rejection event, prednisolone was reintroduced to 10 mg once daily. Rejection was defined as presumed, treated acute rejection (in patients who had no biopsy or an inconclusive biopsy) or biopsy-proven rejection (BPR) as part of routine clinical care by a renal pathologist using 2 μm paraffin sections stained for HE, PAS, Jones and immunohistochemistry for C4d on 4 μm sections. After the completion of the study, all biopsies were reviewed again by a clinical pathologist (M.C.C.) in a blinded fashion per the Banff'15 classification (39). Patients suffering from vascular (Banff grade 2) or tubulo-interstitial (Banff grade 1) rejection received high-dose pulse intravenous methylprednisolon as first-line therapy (methylprednisolone 1,000 mg i.v. for 3 consecutive days), whereas patients suffering from severe or glucocorticoid-resistant acute rejection were treated with alemtuzumab (one or two doses of 30 mg s.c.). Patients suffering from ABMR or mixed-type rejection (TCMR and ABMR) were treated with methylprednisolone plus intravenous immunoglobulins (IVIg) (40, 41). While the majority of patients in our study were treated according to the above mentioned guidelines, there are some instances where biopsy scores were changed after the second biopsy revision done according to the Banff'15 classification. A disparate treatment from the guidelines was also chosen for a few patients for other clinical reasons (e.g., suspicion of humoral immunity components or patient history).

Thirty age and gender-matched healthy controls were also included. All patients (MEC-2016-718, NL59284.078.16) and healthy controls (MEC-2018-1623.; NL66443.078.18) provided written informed consent. The baseline demographic and clinical characteristics of patients and controls are summarized in Table 1.

**Table 1:** Characteristics of kidney transplantation recipients and healthy controls.

	**KTx patients**	**HCs**
**Recipient**		
Number	211	30
Age^a^, years, median (range)	55 (21-77)	55 (25-78)
Male gender, *n* (%)	143 (67.8)	21 (70.0)
**Primary disease**, ***N*** **(%)**		
Hypertensive nephropathy	41 (19.4)	
Polycystic kidney disease	33 (15.6)	
Diabetes mellitus	32 (15.2)	
Glomerulonephritis/pyelonephritis	17 (8.1)	
IgA nephropathy	15 (7.1)	
Focal segmental glomerulosclerosis	9 (4.3)	
Other	38 (18.0)	
Unknown	26 (12.3)	
**Donor**		
Donor age, years, median (range)	54 (0–82)	
Living donor, *n* (%)	155 (73.5)	
**HLA mismatch**, ***n*** **(%)**		
0–2	60 (28.4)	
3–4	92 (43.6)	
5–6	59 (28.0)	
**Induction therapy**		
Anti-CD25 mAb	202 (95.7)	
Rituximab	7 (3.3)	
Anti-thymocyte globulin	2 (1.0)	
**Immunosuppression**, ***n*** **(%)**		
Tac/MMF	181 (85.8)	
Tac monotherapy	30 (14.2)	
Previous KTx, *n* (%)		
Second/third/fourth KTx	19 (9.0)/5 (2.2)/3 (1.3)	
**PRA, mean (range)**		
Current	5.0 (0–83)	
Highest	12.8 (0–100)	
Time after KTx, years, median (range)	5.3 (5.0–7.7)	
**Occurrence of rejection**, ***n*** **(%)**		
No	162 (76.8)	
Yes	49 (23.2)	
First episode within 1Y after KTx	38 (18.0)	
**Rejection type**, ***n*** **(%)**		
TCMR/ABMR/mixed TCMR and ABMR*	29 (13.7)/15 (7.1)/5 (1.4)	

a Age at transplantation for patients. KTx, kidney transplantation; HCs, healthy controls; Pred, prednisolone; Tac, tacrolimus; MMF, mycophenolate mofetil; Anti-CD25 mAb, anti-CD25 monoclonal antibody; Ab, antibody; 1Y, 1 year; TCMR, T cell-mediated rejection; cABMR, chronic antibody-mediated rejection *According to the Banff 2015 classification (39).

### Flow Cytometry

Fresh peripheral blood samples (5–7 years post-transplant) were collected in 6 ml heparin tubes (BD Biosciences, San Jose, CA) and stored at room temperature on a tube-roller. Blood samples were processed within 2 h after drawing blood. Peripheral blood mononuclear cells (PBMCs) were isolated using Ficoll gradient medium (Histopaque-1077, Sigma-Aldrich, St. Louis, MI). In brief, the surface and intracellular staining of PBMCs were performed using the following monoclonal antibodies (mAbs): anti-CD3 BV510 (OKT3, Biolegend, San Diego, CA); anti-CD4 BV421 (RPA-4, Biolegend); anti-CXCR5 AF647 (RF8B2, BD Biosciences); anti-Foxp3 PE (PCH101, Invitrogen, Amsterdam, the Netherlands); anti-PD-1 APC-Cy7 (EH12.2H7, Biolegend); Helios PerCP-Cy5.5 (22F6, Biolegend). The following CD4^+^ T cell subpopulations were studied and defined as: total cTfh (CD3^+^CD4^+^CXCR5^+^Foxp3^−^), total cTfr (CD3^+^CD4^+^CXCR5^+^Foxp3^+^),PD-1^+^cTfh (CD3^+^CD4^+^CXCR5^+^Foxp3^−^PD-1^+^),PD-1^+^cTfr (CD3^+^CD4^+^CXCR5^+^Foxp3^+^PD-1^+^),and Helios^+^cTfr(CD3^+^CD4^+^CXCR5^+^Foxp3^+^Helios^+^). To calculate absolute numbers of CD19^+^, CD4^+^ cells and the above described subsets, BD multi-test 6-color® was used in combination with BD TruCount Tubes® (BD Biosciences). Absolute numbers of CD4 T cell subsets were calculated using the percentages of these subsets within the total CD4 population. Cells were acquired using a BD Canto II flow cytometer and results were analyzed with Kaluza V2.1 software. Gating strategies are shown in **Figure 2**.

### Detection of Anti-HLA Antibodies

The complement-dependent cytotoxicity cross-match was negative before transplantation in all patients. Serum samples from recipients were screened for the presence of HLA antibodies using the Lifecodes Lifescreen Deluxe (LMX) kit, according to the manufacturer's manual (Immucor Transplant Diagnostics Inc. Stamford, CT, USA) 5–7 years post-transplant at the time of blood sampling. Anti-HLA class I (HLA-A, HLA-B, or HLA-C) or HLA class II (HLA-DR or HLA-DQ) antibodies were further analyzed with a Luminex Single Antigen assay using LABscreen HLA class I and class II antigen beads (One Lambda, Canoga Park, GA, USA), as described in our previous study (42). The presence of donor-specific antibodies (DSA) was determined by comparing the various HLA specificities with donor HLA typing.

### Statistical Analyses

Data are presented as median with interquartile range (IQR), unless otherwise specified. The Mann–Whitney *U*-test was used for comparisons between two groups. The Kruskal-Wallis test with Dunn's test for multiple comparisons was used for comparisons between three or more groups. The association between two variables was analyzed by Spearman's correlation and graphically represented by scatter plot. Statistical analysis was performed by IBM SPSS version 25 (Armonk, NY, USA). *P*-values with a two-sided α < 0.05 were considered statistically significant.

## Results

### Characteristics of the Kidney Transplant Recipients

Table 1 depicts the characteristics of the study cohort and healthy controls (HCs). The majority of patients were male (67.8%) and received their first kidney transplant (87.5%) from a living donor (73.5%). Out of 211 patients, 49 patients (*n* = 5 presumed, treated rejection, and *n* = 44 BPR) (23.2%) experienced a rejection of whom 32 recipients (15.2%) experienced one rejection episode, 14 patients (6.6%) had two rejection episodes and 3 patients (1.4%) had three rejection episodes. The majority of patients, 38 out of 49 (77.6%), developed a rejection within the first year after transplantation.

**Figure 1:**
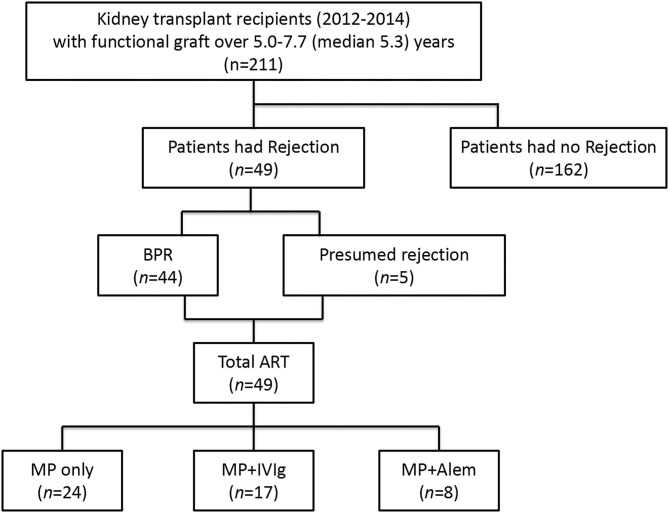
Overview of the kidney transplant recipients with a biopsy-proven rejection before blood sampling and the type of anti-rejection therapy administered. BPR, biopsy-proven rejection; ART, anti-rejection therapy; MP, methylprednisolone; IVIg, intravenous immunoglobulin; Alem, alemtuzumab.

An overview describing the number of patients who developed a rejection and received anti-rejection therapy is given in Figure 1.

### The Effect of Tacrolimus-Based Immunosuppression on cTfr and cTfh Cell Numbers in Kidney Transplant Recipients

**Figure 2:**
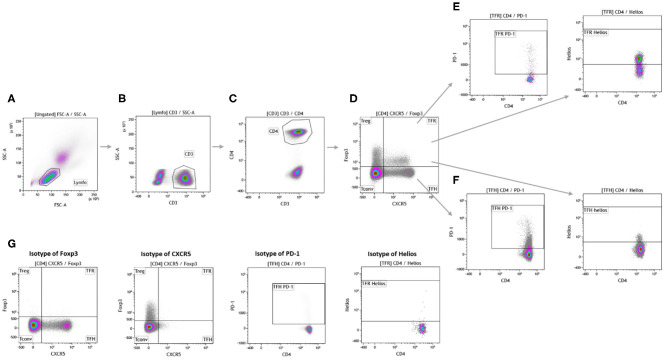
Gating strategy for analysis of circulating follicular T cells. Measurements were performed on freshly drawn blood samples. Lymphocytes **(A)** were gated based on forward and side scatter. CD3^+^ cells **(B)** were used to identify T cells and CD4^+^ cells **(C)** were used for further analysis. Foxp3 and CXCR5 were used to distinguish conventional T cells, regulatory T cells, circulating follicular regulatory T cells, and circulatory follicular helper T cells **(D)**. The expression markers PD-1 and Helios were used to further identify the subsets of circulating follicular regulatory T cells **(E)** and circulating follicular helper T cells **(F)**. Isotype controls were done to ensure proper gating **(G)**.

**Figure 3:**
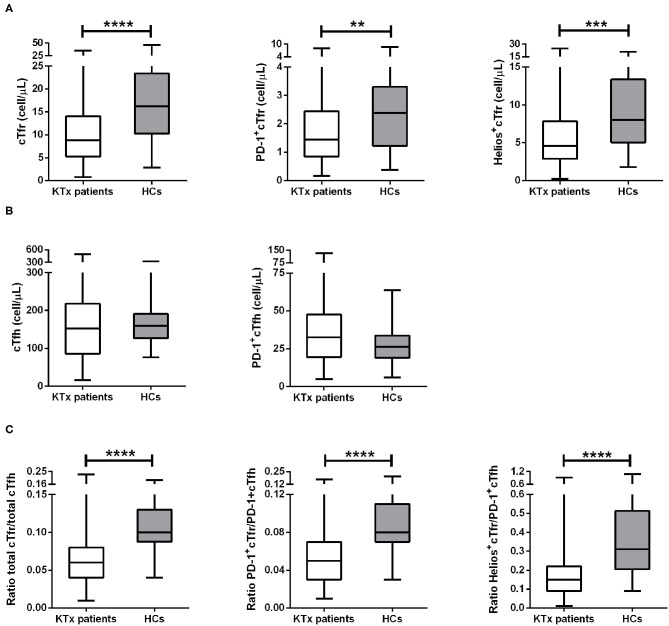
Changes in cTfr and cTfh cell numbers in all kidney transplant patients. The above image shows the absolute numbers (cells/μL) of cTfr cells and its subsets **(A)**, absolute numbers (cells/μL) of cTfh and its subset **(B)**, and cell ratios of cTfr to cTfh and ratios of their subsets **(C)**. Boxplots represent the median and interquartile ranges and the whiskers show the 95% confidence interval. KTx, kidney transplantation; HCs, healthy controls. Mann-Whitney-*U*-test, **p < 0.01; ***p < 0.001; and ****p < 0.0001.

We studied the differences in absolute cell numbers of Tfr and Tfh cells and their PD-1 and Helios expression between kidney transplant recipients and HCs. The gating strategy is shown in Figure 2. The numbers of both total cTfr (CD3^+^CD4^+^CXCR5^+^FoxP3^+^),PD-1^+^cTfr (CD3^+^CD4^+^CXCR5^+^FoxP3^+^PD-1^+^), and Helios^+^cTfr(CD3^+^CD4^+^CXCR5^+^FoxP3^+^Helios^+^) cells were significantly lower in patients when compared to healthy controls (Figure 3A), while the numbers of both total cTfh (CD3^+^CD4^+^CXCR5^+^FoxP3^−^) and PD-1^+^cTfh (CD3^+^CD4^+^CXCR5^+^FoxP3^−^PD-1^+^) in patients and controls were not significantly different (Figure 3B). The decreased cTfr numbers resulted in significantly lower cTfr/cTfh, PD-1^+^cTfr/PD-1^+^cTfh and Helios^+^cTfr/ PD-1^+^cTfh ratios in patients (Figure 3C). Finally, we examined whether the cTfh and cTfr populations were associated with kidney graft function. We found that the number of total cTfr cells, and ratio of cTfr to cTfh were both positively correlated with eGFR (Figure S1). All other cell types showed no significant correlation with kidney graft function.

### Numbers of cTfr and cTfh in Patients With Anti-HLA Antibodies and Donor-Specific Antibodies

**Figure 4:**
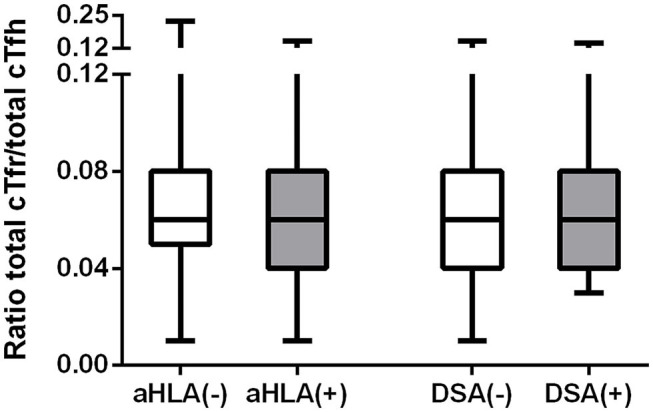
Cell ratios of absolute numbers of cTfr to cTfh in patients with or without anti-HLA antibodies or donor-specific antibodies. The above image shows the ratios of cTfr to cTfh. Boxplots represent the median and interquartile ranges and the whiskers show the 95% confidence interval. aHLA, anti-HLA antibodies; DSA, donor-specific antibodies.

In this study, anti-HLA antibodies were measured for all patients at the time of blood sampling. Out of 211 patients, 34 (16.1%) patients had detectable anti-HLA antibodies, of which 11 (5.2%) were DSA positive. The association between the numbers of cTfr and cTfh cells with the presence of anti-HLA antibodies or DSA was analyzed. No significant differences were observed between absolute numbers of cTfr, cTfh, and cell ratios (Figure 4) in patients with anti-HLA antibodies or DSA at the time of blood sampling.

### cTfr and cTfh in Patients With a History of Rejection and the Effect of Anti-rejection Therapy on Their Cell Numbers

**Figure 5:**
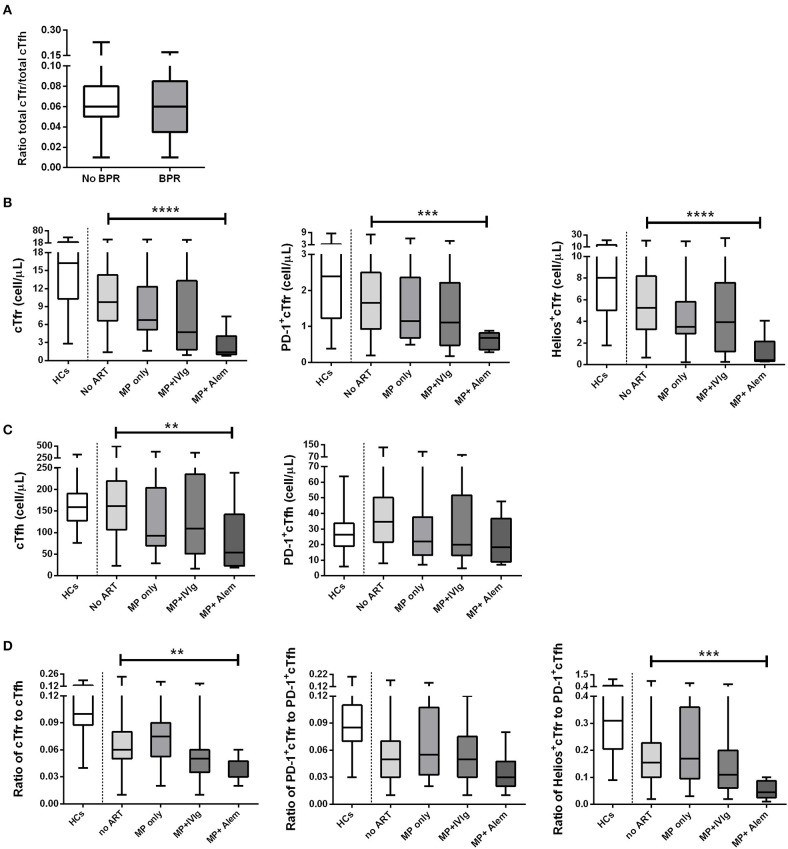
Absolute numbers and ratios of cTfr, cTfh, and their subsets in healthy controls and patients who received different types of anti-rejection therapy. The above image shows the ratio of cTfr to cTfh in patients with and without rejection **(A)**. Also shown are the absolute numbers (cells/μL) of cTfr cells and its subsets **(B)**, absolute numbers (cells/μL) of cTfh and its subset **(C)**, and cell ratios of cTfr to cTfh and ratios of their subsets **(D)**. Boxplots represent the median and interquartile ranges and the whiskers show the 95% confidence interval. HC, healthy controls; ART, anti-rejection therapy; MP, methylprednisolone; IVIg, intravenous immunoglobulin; Alem, alemtuzumab. Kruskal–Wallis with Dunn's multiple comparisons. **p < 0.01; ***p < 0.001; and ****p < 0.0001.

To define whether alloreactivity and subsequent anti-rejection therapy affect cTfr and cTfh cell numbers, we first grouped the samples according to the rejection history and later according to the type of anti-rejection therapy (Figure 1). The 211 patients were divided into a group with a history of rejection (*n* = 5 presumed, treated rejection, and *n* = 44 BPR) and a group with no previous rejection (*n* = 162). Compared to patients without rejection, patients with a history of rejection had 43.9, 39.4, and 39% lower numbers of cTfr, PD-1^+^cTfr, and Helios^+^cTfr cells, respectively. Furthermore, in the rejection patients cTfh and PD-1^+^cTfh cell numbers were 42.8 and 39.2% lower, respectively. Due to the significant decrease of both cTfr and cTfh, all cell ratios in patients with or without a history of rejection were comparable (Figure 5A). Additionally, we examined whether there were any significant differences in absolute cell numbers between patients with different types of BPR. Patients with rejection were categorized into three subgroups according to their latest biopsy results: TCMR (*n* = 29), ABMR (*n* = 15), mixed TCMR and ABMR (*n* = 5). We found no significant differences in cTfr and cTfh absolute cell numbers between patients with different types of rejection (Figure S2).

Patients with a history of rejection received anti-rejection therapy at a median time of 4.9 years before blood sampling. Since there could be a difference in the effects of the various anti-rejection treatments on follicular cell numbers, patients were grouped accordingly. Patients were treated with high-dose pulse intravenous methylprednisolon only, high-dose pulse intravenous methylprednisolon with intravenous immunoglobulin or high-dose pulse intravenous methylprednisolon with alemtuzumab at a median time of 5.1 (range: 2.1–7.1), 2.5 (0.4–6.5), and 5.0 (2.5–6.1) years before blood sampling, respectively. Table 2 provides an overview of the cohort of 49 patients who received anti-rejection therapy before blood sampling. As expected, alemtuzumab treatment had the strongest effect, and it was the only anti-rejection treatment associated with significantly lower numbers of both cTfr and cTfh cells, including their subsets. When compared to patients who had not been treated with anti-rejection therapy, the median absolute numbers of total cTfr in patients treated with previous pulse methylprednisolone decreased by 30.6%, pulse methylprednisolone + IVIg led to 51.2% decrease and pulse methylprednisolone + alemtuzumab led to 85.8% decrease. Absolute numbers of total cTfr, PD-1-cTfr, and Helios^+^cTfr in patients who had received pulse methylprednisolone + alemtuzumab were significantly lower than cell numbers of patients who had received no anti-rejection treatment (Figure 5B). Treatment with other anti-rejection therapies did not result in a significant measurable decrease of total cTfr or activated cTfr absolute numbers compared to no anti-rejection treatment.

**Table 2:** Characteristics of the 49 kidney transplant recipients who received anti-rejection therapy.

	**Anti-rejection therapy**		
	**MP only (*n* = 24)**	**MP + IVIg (*n* = 17)**	**MP + alemtuzumab (*n* = 8)**
Patients with rejection (*n* = 49)			
**Rejection episodes**, ***n*** **(%)**			
One episode (*n* = 32)	20 (40.8)	8 (8.9)	4 (8.1)
Two episodes (*n* = 14)	4 (8.1)	6 (12.2)	4 (8.1)
Three episodes (*n* = 3)	0	3 (6.1)	0
**Rejection type**, ***n*** **(%)**			
TCMR (*n* = 29)	20 (40.8)	4 (8.1)	5 (10.2)
ABMR (*n* = 15)	3 (6.1)	10 (20.4)	2 (4.1)
Mixed TCMR and ABMR (*n* = 5)	1 (2.0)	3 (6.1)	1 (2.0)

*KTx, kidney transplantation; ART, anti-rejection therapy; MP, methylprednisolone; IVIg, intravenous immunoglobulin; TCMR, T cell-mediated rejection; ABMR, antibody-mediated rejection*.

Similarly, absolute numbers of total cTfh in patients treated with previous pulse methylprednisolone decreased by 42.7%, pulse methylprednisolone + IVIg resulted in 32.6% decrease and pulse methylprednisolone + alemtuzumab resulted in 66.8% decrease compared to total cTfh numbers in patients who had not been treated with anti-rejection therapy. This decrease in absolute numbers of total cTfh was only significant for patients treated with pulse methylprednisolone + alemtuzumab, while PD-1^+^cTfh were not significantly lower compared to patients who did not receive anti-rejection therapy (Figure 5C). The ratio of total cTfr to cTfh and Helios^+^cTfr to PD-1^+^Tfh were also significantly lower in patients who received pulse methylprednisolone + alemtuzumab anti-rejection therapy (Figure 5D). We found a similar decrease in CD3^+^, CD4^+^, and T conventional cells in patients treated with pulse methylprednisolone + alemtuzumab anti-rejection therapy. Interestingly, Treg cell numbers were not significantly lower in patients who received pulse methylprednisolone + alemtuzumab anti-rejection therapy (Figure S3).

## Discussion

In the present study, we investigated whether immunosuppression affects the absolute numbers of cTfr and cTfh cells and the balance between cTfr to cTfh cells in kidney transplant recipients. We found that tacrolimus-based maintenance immunosuppression results in decreased absolute numbers of cTfr cells, without affecting cTfh cells when compared to healthy individuals. Other studies in renal transplant recipients, demonstrated increased cTfh cell numbers before the development of DSA, in association with ABMR or with the development of anti-HLA antibodies (43–45). Moreover, decreased numbers of Tfr cells were observed in both kidney transplant tissue and peripheral blood of patients with ABMR (32). The majority of patients in our cohort were on a combined maintenance treatment with tacrolimus and MMF. MMF is a known inhibitor of lymphocyte proliferation (46) and calcineurin inhibitors have an adverse effect on the differentiation and function of regulatory T cells (47–49). Circulating Tfr cells are primarily derived from Tregs (50) so it stands to reason that if Treg differentiation is inhibited then this may also have a direct effect on cTfr cell numbers.

We also found that patients with anti-HLA antibodies or DSA at 5–7 years post-transplant had similar cTfr and cTfh cell numbers as patients without antibodies. This is somewhat unexpected as other studies have shown elevated numbers of cTfh in patients with anti-HLA antibodies or DSA (45, 51). In this study, 13 of the 34 patients with anti-HLA antibodies had a history of rejection and anti-rejection therapy. This suggests that the immunosuppression regimen and/or anti-rejection therapy administered to our patients is able to prevent the expansion of cTfh cells which are commonly associated with the presence of anti-HLA antibodies. However, it may not be sufficient to completely suppress previously formed Tfh and memory B cells from developing anti-HLA antibodies.

As demonstrated in this study, treatment with alemtuzumab led to significantly lower numbers of total and subsets of cTfr and total cTfh cells. After administration of alemtuzumab, T cells repopulation occurs through the clonal expansion of T cells as opposed to repopulation though thymopoiesis, in a process known as homeostatic proliferation (52–54). Our findings suggest that the homeostatic proliferation of cTfr cells is impaired after treatment with alemtuzumab. This is in line with the findings of Macedo et al., who showed that Tregs are significantly decreased compared to HCs several years after alemtuzumab induction (55). Moreover, the resulting shift in balance between Tfr to Tfh cells after treatment with alemtuzumab may result in increased formation of memory B cells and plasma blasts thereby increasing the risk for development of ABMR. Clinical studies have reported that alemtuzumab induction therapy, is associated with a higher incidence of chronic ABMR in kidney transplant recipients (56, 57). It is unclear whether alemtuzumab therapy has an effect on follicular T cells and B cells which reside in SLOs. If these cells remain present in SLOs, T and B cell cross-talk can still result in the formation of memory B cells and antibody-producing B cells. It is also interesting to note that despite successful treatment of rejection, the patients in our cohort have a higher incidence of anti-HLA antibodies and DSA up to 5 years after treatment with anti-rejection therapy.

Nonetheless, the results presented in this study should be interpreted in light of the study limitations. The patient cohort described here is heterogeneous and includes patients treated with different modalities of anti-rejection therapy and differences in the timing of treatment. While this heterogeneity is representative of daily clinical practice, it provides a challenge when interpreting our results. Another limitation is the fact that cell counts prior to treatment with anti-rejection therapy are not available. Ideally, serial measurements both before and after treatment with said therapies would provide interesting data. As comparisons with pre-treatment samples are not possible to conclude with certainty whether cTfh cell numbers are less affected by anti-rejection therapy or whether there is a faster repopulation of this subset compared to that of cTfr cells. And finally, the challenges in acquiring sufficient numbers of follicular T cells in transplant recipients results in a lack of assays to describe the functionality of these cells after treatment with maintenance and anti-rejection therapy. The use of a mouse animal model might be valuable method to study the mechanisms of follicular T cells *in vivo*. Moreover, by using a mouse monoclonal antibody against the CD52 molecule it would also be possible to study the effect of alemtuzumab on follicular T cells and B cells present both in the circulation and in SLOs.

In conclusion, compared to healthy controls, numbers of cTfr cells in peripheral blood of kidney transplant recipients on tacrolimus-based therapy are significantly lower up to 7 years after transplantation. Furthermore, previous treatment with alemtuzumab has a significant and long-lasting effect on the numbers of these cells.

## Data Availability Statement

The raw data supporting the conclusions of this article will be made available by the authors, without undue reservation.

## Ethics Statement

The studies involving human participants were reviewed and approved by METC Erasmus Medisch Centrum Rotterdam. The patients/participants provided their written informed consent to participate in this study.

## Author Contributions

QN, AM, CB, NB, TG, and DH: conceptualization. MD and CB: methodology. MD, QN, and AM: investigation. QN and AM: formal analysis. QN, AM, and CB: writing-original draft. LW, NB, TG, and DH: editing. NB and CB: supervision. MD and NB: project administration. All authors contributed to the article and approved the submitted version.

## Conflict of Interest

The authors declare that the research was conducted in the absence of any commercial or financial relationships that could be construed as a potential conflict of interest.

## Supplementary Material

Figure S1 Correlations of cTfr absolute cell numbers **(A)**, cTfh absolute cell numbers **(B)**, and ratio of cTfr to Tfh **(C)** with kidney function. Spearman's correlation.

Figure S2 Absolute numbers and cell ratios of cTfr, cTfh, and their activated subset by type of rejection. Shown are the absolute numbers (cells/μL) of cTfr cells and its subsets **(A)**, absolute numbers (cells/μL) of cTfh and its subset **(B)**, and cell ratios of cTfr to cTfh and ratios of their subsets **(C)**. Boxplots represent the median and interquartile ranges and the whiskers show the 95% confidence interval. TCMR, T cell-mediated rejection; ABMR, antibody-mediated rejection; Mixed, mixed TCMR and ABMR. Kruskal–Wallis test.

Figure S3 Absolute numbers of CD3, CD4, T conventional cells and Treg cells in healthy controls and patients who received different types of anti-rejection therapy. The above image shows the absolute numbers (cells/μL) of CD3 cells **(A)**, CD4 cells **(B)**, T conventional cells **(C)**, and Treg cells **(D)**. Boxplots represent the median and interquartile ranges and the whiskers show the 95% confidence interval. HC, healthy controls; ART, anti-rejection therapy; MP, methylprednisolone; IVIg, intravenous immunoglobulin; Alem, alemtuzumab. Kruskal–Wallis with Dunn's multiple comparisons. ^*^*p* < 0.05 and ^**^*p* < 0.01.

## Abbreviations

Alem: alemtuzumab
ART: anti-rejection therapy
BPR: biopsy-proven rejection
CNIs: calcineurin inhibitors
cTfh cell: circulating follicular helper T cell
cTfr cell: circulating follicular regulatory T cell
DSA: donor-specific antibodies
IVIg: intravenous immunoglobulin
KTx: kidney transplantation
MMF: mycophenolate mofetil
MP: methylprednisolone
PBMC: peripheral blood mononuclear cells
Pred: prednisolone
Tac: tacrolimus
TCMR: T cell-mediated rejection.
